# Seasonal workers wanted! Germany’s seasonal labour migration regime and the COVID-19 pandemic

**DOI:** 10.1177/02633957231168039

**Published:** 2023-05-08

**Authors:** Dorothea Biaback Anong

**Affiliations:** Humboldt-Universität zu Berlin, Germany

**Keywords:** Covid-19, logistification, migration, migration regime, seasonal work

## Abstract

The COVID-19 pandemic publicly exposed the urgent need for seasonal workers in agriculture. In Germany, an entry ban and entry quotas for seasonal workers at the beginning of the pandemic caused major attention. Taking this moment as magnifying glass, the article asks how the German seasonal labour migration regime is constructed (legally) and legitimated (discursively), and in how far the pandemic has caused shifts within this regime. Based on an analysis of the legal framework and the political discourse around seasonal work from 2018 to 2020 in Germany, the seasonal labour migration regime is characterised as just-in-time migration tailored to the needs of agricultural business, where migrants’ work force is not absorbed homogenously by precarious labour sectors, but rather specific groups of migrant workers are integrated differently through mechanisms of differential inclusion. Within this regime, seasonal workers function as outsourced labour, whose reproduction costs remain abroad. On the discursive level, the article shows how seasonal workers are produced as ‘wanted migrants’ by linking seasonal migration to the interests of the ‘homeland’. While the pandemic momentarily caused some shifts on the discoursively level, the article shows that the seasonal labour regime as a whole remains rather stable in time.

## Introduction

‘Corona Crisis: Asparagus farmers urgently looking for harvest helpers’

—(Terpitz, 2020)

At the beginning of the COVID pandemic in spring 2020, similar cover stories dominated German newspapers. A travel ban for seasonal workers pronounced in late March and the resulting shortage in the labour force for asparagus harvesting made them a major issue of public and political debate, peaking in an internationally debated exceptional monthly entry permit for 40,000 agricultural workers, who were flown across closed borders on charter flights. This intense public attention stood in sharp contrast to the prior status quo, when seasonal migrant workers in Germany had been ‘nearly invisible to society’ (Becker, 2010: 12).^1^ I study the pandemic as a moment of crisis, when the disruption of the migrant labour supply suddenly highlighted the long-standing but publicly overlooked dependency on a migrant seasonal work force in German agriculture. After the travel ban was lifted and the exceptional regulation expired in June 2020, the public and political attention quickly ceased. However, this moment of visibility and society’s sudden confrontation with the meaning of seasonal workers for food supply, which triggered the political need to legitimate the exceptional measures taken, served as a magnifying glass uncovering formerly implicit logics within the regime of seasonal labour migration. To unpack this regime, I conducted an analysis of the political discourse around seasonal labour migration in German agriculture from January 2018 to August 2020, as well as of the legal regulations and political measures before and during the pandemic. Strikingly, in contrast to other discourses around migration and migrant workers, throughout the study period, all political actors under investigation were in favour of seasonal labour migration. Even the neo-right-wing and nationalist party AfD (Alternative for Germany), which fundamentally opposes migration to Germany, argued for the increased and facilitated entry of seasonal workers. Starting from this puzzle, I investigate the German seasonal labour migration regime before and during the pandemic, asking how it is (legally) constructed to facilitate this specific form of labour migration, how seasonal labour migration is politically produced as wanted migration, and to what extent the pandemic has induced changes within the regime.

In Germany, parallel to the invisibility of seasonal workers in society, migrant labour in agriculture has been studied only scarcely (for exceptions, see Becker, 2010; Carlotta von Bock und Polach, 2011; Wagner and Hassel, 2015). This changed slightly during the pandemic. The pandemic related works mostly focus on worsened working conditions and workers’ organising against exploitation (Birke, 2020; Bogoeski, 2022; Cosma et al., 2020). In other geographical contexts, like Canada or Southern Europe, an extensive body of literature documents the exploitation of migrant labour in agriculture, including a growing number of articles related to COVID19 (e.g. Bejan et al., 2021). Particularly (but not only), earlier contributions draw on macro-economic theories like labour market segmentation theory (Piore, 1979) or the Marxist idea of migrants as the new industrial reserve army (Castles and Kosack, 1972) to explain the high dependency on a temporalized migrant work force in agriculture (e.g. Basok, 2002; Martínez Veiga, 1998; Rye and Scott, 2018). Even though these macro theories on the political economy of labour migration are well fit to grasp the basic role of seasonal labour migration in agriculture within the international division of labour, they need to be refined to address the specific and fine-tuned processes that produce and maintain seasonal labour migrants as an exploitable labour force in current migration regimes.

Therefore, in this article I zoom into the government of seasonal labour migration in Germany in the context of the pandemic to provide a more fine-grained analysis of the logics and mechanisms that (re)produce and legitimate the exploitation of seasonal migrant workers. I show, how the seasonal labour migration regime is tailored to the needs of agricultural business in terms of a *just-in-time migration* (Altenried et al., 2017). Migrants’ work force is not absorbed homogenously by precarious labour sectors, but rather mechanisms of *differential inclusion* (Mezzadra and Neilson, 2013) are at play, which integrate specific groups of migrant workers differently by stratifying access to rights. Situating this regime within the political economy of labour migration, I understand seasonal workers as *outsourced labour* (Mezzadra and Neilson, 2008), whose reproduction costs remain abroad. On the discursive level, I demonstrate how seasonal labour migrants are produced as ‘wanted migration’ serving national interests.

In the following, I provide some general information on seasonal work in German agriculture for context, to then outline my research design. After a short discussion of common theoretical approaches to seasonal labour migration, the analytical part presents how the German seasonal labour migration regime is (legally) constructed and discursively produced and the changes induced (or not) by the pandemic. I end with some concluding remarks on the current regime and its future.

## Status quo of seasonal work in German agriculture before the pandemic

About 300,000 foreign workers enter Germany for seasonal work in agriculture every year. The German Statistics Office officially counted 286,300 employees in 2016 and 274,400 in 2020 (Destatis, 2021). Even though no reliable statistical data is available, it is estimated that foreign labour migrants make up at least 95% of seasonal workers in German agriculture (German Farmers’ Association (DBV), 2019). While, traditionally, Polish migrants made up the majority, during the last decade Romania has become the main country of origin (Wagner and Hassel, 2015: 8).

Citizens from outside the European Union (EU) generally cannot work legally as seasonal workers in Germany. In 2014, the EU adopted the *Seasonal Workers Directive*, that allowed the member states to enter bilateral agreements on seasonal labour migration with other states (European Union, 2014). However, it was not until May 2021 that Germany started a bilateral pilot project with Georgia for the recruitment of 5000 seasonal workers (Lechner, 2021: 5) and 1 year later with the Republic of Moldova (Federal Labour Office, 2022). This is why seasonal workers in German agriculture generally come from other EU countries. There are no reliable data on undocumented seasonal work in German agriculture (European Migration Network (EMN), 2017). While it undoubtedly exists, the experiences of counselling services and results from customs inspections suggest that irregularity mostly concerns infringements against the minimum wage and social security regulations rather than work without a permit (dpa, 2020).

As seasonal labour migration in German agriculture is mostly undertaken by EU citizens who enjoy access to the German labour market under equal conditions with nationals, the most important regulations are no longer determined by migration policy measures but by general German labour law. Most relevant are the provisions on short-term employment, which is exempt from social security contributions, and on the minimum wage. Formerly, a short-term employment could be exempt from social security, if it did not exceed the maximum of 50 working days. In 2015, this period was increased to 70 days. While at that point, the extension was meant to be temporary, at the beginning of 2019, it was declared permanent. In practical terms, the exemption from social security means that seasonal workers do not acquire pension rights and that they are not covered by public health insurance. In 2015, a minimum wage of 8.50 Euro was introduced in Germany (which has risen to 9.82 Euro by 2022). Given that before, wages between 5 and 7 Euros were common for seasonal workers (Gesamtverband der deutschen Land- und Forstwirtschaftlichen Arbeitgeberverbände (GLFA), 2019), agricultural businesses and advocacy groups presented a strong opposition to the minimum wage for seasonal work.

## Discourse-oriented analysis of the seasonal labour migration regime

Following Vasilis Tsianos and Karakayali (2010: 375), this article departs from a concept of migration regime that understands ‘regulations of migration as effects, as condensations of social actions instead of taking regulations functionalistically for granted’. This notion of a regime rather implies ‘a space of negotiating practices’ (Tsianos and Karakayali, 2010) constituted by ‘diverse actors, forces, discourses, interests, and economies’ (Tsianos and Karakayali, 2010: 378).

I am particularly interested in the systemic content of regimes ‘that can be located precisely in those “questions and problems” that different actors are negotiating amongst each other’ (Georgi, 2016: 187). This perspective is informed by Bob Jessop’s (2016: 53) concept of the state and state power as ‘an institutionally and discursively mediated condensation (a reflection and refraction) of a changing balance of forces’. Building on a Gramscian concept of hegemony, I share Jessop’s (2016: 72) assumption that the socially recognised legitimacy of state action depends on the extent to which certain decisions and measures can plausibly be presented as ‘common sense’ to the population. For my analysis, the combination of the (migration) regime approach with Jessop’s capitalist state theory makes it possible to study discursive political negotiation processes as struggles for hegemony as well as the specific actors and regulatory practices (in form of the legal framework, regulations, and political ad hoc measures taken during the pandemic) as central building blocks of the migration regime. This perspective entails a focus on governmental logics and (discursive) practices. While I agree with other authors that migrants’ practices are a constitutive part of migration regimes – as they inform as well as react to governmental practices (Scheel, 2017) – these have not been the object of this empirical research.

To identify the specific argumentation patterns political actors apply to legitimise their practices or demands as ‘common sense’, a discourse-oriented argumentation analysis following Martin Wengeler (2003) was conducted. The method is based on the so-called topoi, that is, argumentation patterns ‘whose conclusiveness or conclusion follows from premises that are [socially] accepted opinions’ (Römer, 2018: 122). In detail, roughly, 600 single argumentations have been aggregated into main topoi by identifying common underlying premises, which are explicated in italic below. By interrelating these argumentation patterns with the legal framework and state actions regarding seasonal work, this methodological approach makes it possible to empirically trace the governmental logics and political reasoning within the German seasonal labour migration regime. The COVID pandemic as a moment of crisis was crucial to the identification of these argumentation patterns. The sudden visibility of seasonal workers in agriculture obliged decision makers to explicitly legitimise not only the political action concerning seasonal work migration taken during the pandemic, but also the status quo of the seasonal labour migration regime.

The analysis included publications by the central political institutions involved in policy making regarding seasonal work (namely, the German Parliament, the Federal Government, and the following ministries: Ministry of Agriculture, Ministry of the Interior, Ministry of Labour and Social Affairs) as well as by the ‘German Farmers’ Union’ (DBV) and the workers’ union for the building sector and agriculture (IG BAU) representing the most important advocacy groups for employers’ and workers’ interests. All available publications were scanned for the words ‘harvest’ and ‘seasonal’, and included into the analysis after eliminating documents not addressing seasonal work in agriculture. As for the time frame, I chose January 2018 as a starting point to include the main political debate around seasonal work prior to the pandemic, which arose around the question of social-security exemptions (see above), while in August 2020, the debate around seasonal work triggered by the COVID outbreak had mostly subsided and data collection was therefore closed.

The inductive analysis identified the following main topoi (listed with the associated socially accepted premises they build on): the ‘economic needs’ topos (*There is an economic need for seasonal labour, therefore, certain political measures are to be supported/rejected*), the ‘supply’ topos (*The food supply of the German population must be ensured, therefore, certain political measures are to be supported/rejected*), the ‘working conditions’ topos (*Certain political measures have a positive/negative impact on the working conditions of seasonal workers and are therefore to be supported/rejected*), and the ‘suitability’ topos (*A certain group is (not) suitable to do seasonal work in agriculture, therefore they should/can (not) be activated for this work*). The topoi ‘economic needs’ and ‘supply’ often appeared in connection with arguments that focussed on the ‘homeland’ and safeguarding German national interests, while for the topos of ‘suitability’, arguments relating to the qualification of seasonal workers were particularly relevant.

## The politics of (re)producing an exploitable seasonal labour force

Theoretically, seasonal work migration has mostly been analysed pointing to economic theories of migration, most importantly segmented labour market theory. It centres around the observation that upwards mobility of domestic workers causes a shortage in labour force for low-wage and precarious jobs, which is filled by migrant workers. Developed against the backdrop of state recruitment programmes for so-called guest workers in post-war Western Europe, this theory conceptualised migrants as ‘target earners’ (Piore, 1979: 50), assuming migrants’ filling of labour shortages in the secondary labour market without posing claims to social inclusion to be a general function of labour migration. Jobs in this ‘secondary labour market’ are marked by high labour intensity and fluctuations in labour demand, which can be mitigated by migrant labour. In a similar vein, Castles and Kosack (1972) conceptualise migrants as the ‘new industrial reserve army’ in Marxist tradition, buffering capitalist crises. I agree with the aforementioned authors that these theories can still grasp the general function of seasonal migrant workers for labour markets in high-income states, filling precarious but essential job positions, nationals are not available for. However, beyond this macro-economic statement they cannot account for the politics of regimes enabling and perpetuating this form of international labour division serving domestic labour needs. Therefore, in the following I elaborate how the German seasonal labour migration regime is constructed (legally) and legitimated (discursively), and in how far the pandemic has caused shifts within that.

### Seasonal labour migration as just-in-time migration

According to Altenried et al., migration management is increasingly guided by logistical principles. Specifically, they observe a tendency in which migration management aims to ‘place exactly the right amount of labour, with the right qualities (e.g. their qualifications), at the right time and in the right place’ (Altenried et al., 2017: 54). They therefore describe the emergence of a *just-in-time* and *to-the-point* migration, referring to the logistical ‘guiding principle of just-in-time and to-the-point production’ of goods (Altenried et al., 2017: 7). For seasonal labour migration in German agriculture, the temporal aspect of tailoring the regime to provide the specific amount of migrant labour force for the specific period of time needed in agriculture is particularly relevant and insightful. Therefore, I use the abbreviated form *just-in-time migration* here.

Several political measures show, how this tailoring peaked at the beginning of the pandemic. Shortly after the entry ban for seasonal workers pronounced in March 2020 – which was followed by an immediate outcry by German crop farmers due to the expected labour shortages for the starting harvesting season – several measures were announced to secure the agricultural workforce. Only 2 days later, the social security exemption for seasonal work was temporarily extended to 115 days (German Ministry of Labour and Social Affairs, 2020a), so that migrant workers who were already in the country could work longer without incurring higher labour costs. In addition, agriculture was included into the newly regulated set of ‘essential’ sectors, where the maximum of allowed working hours was extended from the general legal maximum of 8 daily and 40 weekly hours to 12 and 60 hours, respectively (German Ministry of Labour and Social Affairs, 2020b). One week after the entry ban, the government decided on an entry quota of 40,000 agricultural seasonal workers per month, even though the borders remained closed for other workers (German Ministry of the Interior/German Ministry for Agriculture, 2020).

According to the German Ministry for Agriculture (2020d), the extended social security exception for short-term employment to 115 days covered ‘the whole harvesting season until October’, including the two labour intensive periods in spring and autumn. Thereby, the regulation assured that the work force would be available for the exact amount of time needed without incurring additional labour costs.^2^ In addition, the entry quota of 40,000 workers per month met exactly the demanded work force communicated by the farmers’ advocacy group DBV at the beginning of the pandemic.^3^ In combination with the extended working hours, these three regulations made the ‘right’ workers (foreign seasonal workers in contrast to unsuitable domestic workers) maximally available to do the surplus work under the given circumstances. In addition, the quota regulation included a ‘working quarantine’, with workers allowed to work but not to leave the farm in the first weeks after their arrival.

Thus, with regard to seasonal workers, the ultimate migration policy goal of ‘a tailor-made immigration’ (Altenried et al., 2017: 55) fitting the needs of the labour market appears to become real at the moment of the entry quota. In this sense, Peter (Birke, 2020) writes that the ‘radicalised [Covid] border regime with its total blocking of all entries and exits and the “allowing” of quotas for harvest workers [is] approaching the linkage of work and stay in an almost dystopian way’ (Birke, 2020: 10).

This culmination quickly passed after the entry ban and the quota system expired. The adaptation of the legal framework to the specific demands for labour force in agricultural businesses, however, is not an exception due to the crisis, but proves to be part of a continuity. As stated above, since EU citizens from the new EU member states can be employed as seasonal workers in Germany without permission, seasonal work migration is regulated mostly by the legal framework on short-term employment. Thus, general provisions on short-term employment were already amended in 2015 (and then confirmed in 2018/2019) to allow the longer employment of seasonal migrant workers without additional costs for the employers. In January 2019, for instance, the Federal Government justified the renewal of the 70-day regulation with the relief of crop farms that ‘rely on seasonal workers for exactly the 70-day period’ (German Federal Government, 2019: 3).

### Mechanisms of differential inclusion

As I have pointed out before, Piore’s theory of migrants filling unwanted jobs in the secondary labour market can well account for the dependence on exploitable migrant labour in agriculture. It cannot, however, grasp the differentiated mechanisms applied to mobilise certain groups of migrants for this work, which I lay out in the following. These can be described more accurately as processes of *differential inclusion*, in the sense proposed by Sandro Mezzadra and Brett Neilson (2008), through which different groups of migrants are selectively included into society and (precarious) work producing a ‘differential system of filtering and stratification’.

With the entry ban for seasonal workers in place, for a short period of time, the domestic population appeared as the only available labour pool for agricultural work. Therefore, to mobilise an alternative workforce from within the country, contact platforms for companies looking for domestic workers were set up and financial incentives were offered for taking up work in agriculture. These measures did not mobilise significant numbers of local workers to fill the gap. However, they did trigger a new debate on the question, who should fill the shortage of workers in agriculture.

The shortage in seasonal labour force in agriculture did not start with the pandemic. Throughout the investigation period, farmers and the farmers’ union complained about an increasing seasonal labour shortage and difficulties to recruit sufficient workers as an economic challenge for their businesses. While during the 1990s and last in 2005, the German government launched campaigns to activate nationals for seasonal work in agriculture, ever since it had been accepted as an implicit fact that ‘Germans are not available for seasonal work’ (DBV, 2018) and attempts to mobilise seasonal work force had been limited to foreign countries. Considering the increasing difficulties to recruit seasonal workers from other EU countries under the given circumstances and wages, recently the search for alternative seasonal labour was focussed mainly on possible recruitment agreements with countries outside the EU. With the entry ban and the failed attempt to substitute foreign workers with nationals, the reasons why Germans were neither willing nor considered suitable for seasonal work in agriculture became explicit in the political discourse. A part from low wages and the reluctance to work long hours, especially the physical hardship of agricultural labour and nationals’ lack of practical experience were named as reasons, why they were not suitable for this kind of work in contrast to foreign seasonal workers:

It must be borne in mind: Some [national workers] are only available three days a week, [. . .] others have physical complaints. (German Ministry for Agriculture, 2020c: 2).

However, the solutions debated and political measures taken during the first month of the COVID pandemic also showed, how certain groups of migrants were addressed differently to fill the labour shortage. In view of the acute shortage of labour in agriculture, only few days after the entry ban the Federal Labour Office issued a temporary work permit again until October 2020 – for certain groups of refugees and other nationals from countries outside the EU residing in Germany (Ambrüster, 2020). The preceding political discussion was dominated by the debate on a temporary lift of the work ban for certain groups of refugees to let them work in agriculture. Especially, asylum seekers from so-called ‘safe countries of origin’ were believed to have a ‘special interest’ (German Ministry for Agriculture, 2020a: 19–20) in such an exceptional permit to work in agriculture, as this group is generally banned from access to work. Furthermore, unemployed refugees and asylum seekers with work permits should be mobilised because they were considered an ‘immediately available workforce’ (German Federal Government, 2020b: 20).

This moment can well be analysed in terms of differential inclusion in so far, as both asylum seekers and recognised refugees (the latter are entitled to the same labour rights as nationals) are addressed as a potential labour force separate from the rest of society. In contrast to the debate on the mobilisation of national workers, physical strain and relatively low wages are not mentioned as relevant barriers for this group. Therefore, they are expected to be available more easily than the rest of society. Also, contrary to demands from the Greens and Left parties, the temporary working permit did not include the option to gain a residence permit or pension entitlements through this work, nor any options for posterior working permits or working options in other positions. Therefore, the group addressed by this decree were differentially included solely as temporary precarised work force in agriculture. But also among the group of asylum seekers differentiations became visible: while those from ‘safe countries of origin’ were singled out as particularly interested and available for agricultural labour in the political debate, the temporary work permit pronounced on 2 April excluded them from the group of third-country nationals who were allowed to take up employment in agriculture (Ambrüster, 2020). As various authors have pointed out, the restriction of labour and other social rights is part of a political strategy to deter certain groups of asylum seekers who have little ‘prospects of staying’ (Scherschel, 2016: 257) – especially asylum seekers from ‘safe countries’.

With regard to the inclusion or exclusion of EU migrants in Germany on the other hand, Lisa Riedner (2017) has shown that although they have a fundamental right of residence as EU citizens, the exclusion from welfare state benefits restricts the immigration of low or unskilled EU migrants, particularly from South-East Europe. Similarly, other authors state that especially ‘low-skilled migrants from Bulgaria and Romania’ are ‘not wanted’ (Friedrich and Pierdicca, 2014: 133). These exclusionary discourses and practices are highly conflated with racist stereotypes against Roma (Neuburger and Hinrichs, 2022). Against this background, the statement about mostly Romanian seasonal workers made by an MP in parliament, that ‘it is not the case that they want to register any claims to the German welfare system as quickly as possible’ (CDU/CSU, 2018b: 23) appears as a direct juxtaposition to other migrants from the same region, who are considered unwelcome because of their (potential) claims to social welfare. ‘Low-skilled EU migrants’ are only welcome as long as they take on work in the low-wage sector, but leave after their work is done without claiming social security or the right to stay. Thus, EU citizens from South-East Europe are included as workers entitled to free movement whose labour force is available without the bureaucratic and legal restrictions that apply when employing third-country nationals. Beyond the contribution of their labour force, however, their inclusion as permanent migrants is jeopardised by the exclusion from welfare benefits.

The analyses of mechanisms of differential inclusion shows, how the German migration regime welcomes certain groups of ‘low-skilled’ migrant workers (seasonal workers from EU states and third countries or certain asylum seekers) who are temporarily exploitable as labour in the low-wage sector, while remaining largely excluded from social rights. For others, however, a politically motivated logic of deterrence prevails (permanent EU migrants, asylum seekers from ‘safe countries’).

### Seasonal labour migration as outsourced labour

Proposing an influential thesis on the function of migrant labour in capitalist economies Michael Burawoy (1976: 1053) stated that by recruiting labour migrants ‘a proportion of the costs of renewal [of the labour force] is externalized to an alternate economy and/or state’. Building on this observation, Mezzadra and Neilson (2013) have described the circumvention of these social reproduction costs in capitalist states through labour migration with their concept of *outsourced labour*. They state that the selective filtering function of borders does not only create an international division of labour between ‘North’ and ‘South’ (or ‘centre’ and ‘periphery’), but also produces precarised migrant workers as ‘outsourced labour within national territory’ (Mezzadra and Neilson, 2013: 82). Thus, certain migrants work as ‘a kind of supplement to the stock of labour power present within the bounded space of the national labour market’ (Mezzadra and Neilson, 2013: 102). Their labour power is available on the labour market in the state of arrival, while the costs of their social reproduction remain at their country of origin.

Seasonal labour in German agriculture is outsourced in the sense that the workers do not acquire any pension entitlements or other social rights in Germany as a result of their almost exclusively social security–free employment. In the sense of *just-in-time* migration, the legal regulations on short-term employment are being adapted, so that, migrant workers can work in Germany the exact amount of time they are needed on the agricultural labour market. Due to the temporary concordance of their stay to the working period, they stay excluded from the welfare state and the site of social reproduction (social welfare, education, care work, etc.) stays abroad. In this ‘temporalization’ (Altenried et al., 2017: 54) of the migrant work force, the employers’ interest in low-cost labour force coincide with the national interest in the exclusion of migrant workers from the welfare state. In the German political discourse around seasonal migration, this is exemplified, for instance, in the statement cited above that seasonal workers do not ‘want to register any claims to the German welfare system’; or when the right-wing party AfD (2020a: 1) compares seasonal workers ‘who bring great benefit to our population’ to the entry of asylum seekers that would supposedly cause ‘the collapse of the welfare state’.

It is important to note that this ‘highly effective labour migration system’ (Becker and Heller, 2002: 86) of seasonal work in agriculture can only be maintained because the interests of seasonal workers themselves partially coincide with the exception from non-wage labour costs and the temporalisation of stay. Acting strategically within the unequal setting of international wage differentials and limited options for labour mobility (e.g. due to EU workers exclusion from welfare benefits), seasonal workers’ interest to attain the maximum net wage during limited but recurrent work periods:^4^ much in the sense of Piore’s (1979: 50) ‘target earners’: creates higher acceptance of harsh working conditions and (for national standards) low wages in the secondary labour market.

However, as the chronically increasing labour shortage in agriculture already prior to the pandemic shows, this conjunction of interests within the system of outsourced labour is changeable. After Polish labour migrants had turned to more attractive sectors and destinations following the EU Eastern enlargement in 2004, recently, also EU citizens from Romania and Bulgaria are less and less willing to do seasonal agricultural work in Germany under the given conditions and wages. The search for alternative labour reservoirs to maintain this system of outsourced labour therefore increasingly focusses on bilateral agreements with third countries. Due to the higher wage gap, and also the stronger link between residence and work, it is assumed that seasonal migrant workers from non-EU countries are more willing to accept the given working conditions (Initiative Faire Landarbeit, 2020: 18–19).

## The discursive production of seasonal workers as ‘wanted migrants’

From a differentiated political economy perspective, I have argued above that seasonal labour migration functions as outsourced labour in the secondary labour market based on mechanisms of differential inclusion and because of its temporary nature as *just-in-time*-migration. Apart from the political measures and legal regulations aimed at tailoring the seasonal work regime to domestic labour needs, the pandemic has also shown how this regime is discursively legitimated and maintained. Most importantly, the analysis showed how seasonal labour migration is discursively linked to the interests of the nation in terms of the maintenance of the German food production on German soil. Focussing on the moment of the pandemic two discursive shifts can be observed: from representing seasonal workers as ‘helping hands’ to skilled workers, and an intensified political debate around seasonal workers’ working conditions.

The arguments in favour of seasonal work migration in agriculture presented by conservative and right-wing actors within the political discourse mostly referred to the ‘homeland’ and German national interests. The discursive linkage of agricultural seasonal work migration to ‘the homeland’ and German interests occurs through two central patterns of argumentation. On one hand, the necessity of foreign seasonal work for safeguarding the interests of the German population in sufficient food supply, the consumption of certain seasonal vegetables and the maintenance of ‘German’ and ‘domestic’ quality in food production is emphasised. While before the pandemic the conservative party stated: ‘It is important for all of us that good German quality is put on the table’ (CDU/CSU, 2018a: 53), during the corona crisis political measures were justified with the maintenance of ‘high quality domestic food production’ for the ‘German population’ (German Federal Government, 2020c: 88; CDU/CSU, 2020: 2; AfD, 2020b: 2).

On the other hand, the economic interests of the farms are tied to national interests by discursively linking farmers to the ‘homeland’ in terms of the German soil. With regard to the entry quota for seasonal workers in the face of farmers’ economic hardships the German Minister for the Interior stated, for example: ‘The Federal Minister of the Interior nevertheless understands the concerns of the farmers: after all, he is the Minister for the ‘Homeland’’ (German Ministry of the Interior, 2020b: 17). In this nationalist discourse about German high-quality food production by German farmers on German soil, foreign seasonal migrants whose work produces these goods become invisibilised and their work appropriated, not only in economic, but also in symbolical terms. This appropriation is mirrored in the recognition of the agricultural sector as ‘essential’ in the context of the pandemic. By framing agricultural businesses as ‘essential’ for the populations’ food supply (under the ‘supply topos’) the economic interests of the companies appear to be in the interest of the population as a whole in overcoming the crisis.^5^

In order to supply consumers with sufficient food even during the Corona pandemic, the cooperation of foreign seasonal workers [. . .] is absolutely necessary for many farmers (German Federal Government, 2020c: 87).

Measures to relieve the (economic) crisis for agricultural producers, such as the extension of permissible working hours for seasonal workers in agriculture and the extension of social security exemptions, then do not appear to be motivated by purely economic interests of the agricultural sector, but as necessary emergency measures in the face of an extraordinary crisis for the whole population. Thereby, the interests of the ‘homeland’ merge with the idea of economic utility.

While this linkage between the ‘homeland’ and the economic needs of farmers becomes particularly apparent in the moment of a perceived crisis in national food supply, as an argumentation pattern it persists throughout the entire period under study. On the contrary, the pandemic marks a turning point for the way political actors refer to seasonal workers. While in 2018 and 2019, seasonal work in agriculture was generally characterised as ‘helpers’ jobs’ (e.g. DBV, 2018: 1), during the pandemic, seasonal workers were explicitly addressed as ‘skilled workers’ whose skills could not be replaced and whose entry was therefore indispensable for the German population’s food supply:

In order to supply consumers with sufficient and high-quality domestic food during the Corona pandemic, farmers depend on foreign seasonal workers–these are skilled workers (German Ministry for Agriculture, 2020b: 2).

In Germany, as well as in the EU and other high-income countries, current policies of legal labour migration are predominantly designed to promote the entry of high skilled workers. An emblematic testimony to this is the German ‘Skilled Workers Immigration Act’ passed in 2019, which defines a ‘skilled migrant’ as ‘a foreigner who holds a qualified vocational qualification or a [. . .] a university degree’ (§18 Absatz 3 AufenthG). The designation of seasonal workers without formal education as ‘skilled workers’ during the Corona pandemic seems to be at odds with the aforementioned definition. The qualifications presented as specific characteristics of foreign seasonal workers do not refer to professional training. Rather, they paraphrase the characteristics of workers needed in agriculture: practical experience as well as the ability and willingness to perform physically demanding work. As I pointed out at the beginning, low-skilled seasonal workers have always been needed in German agriculture but have been rather invisible to the German society. To legitimise the exception for their entry during the pandemic on the contrary, they had to be marked as part of the migration that is necessary for Germany’s prosperity and publicly recognised as such: skilled workers. Thereby, pointing back to the seasonal labour migration regime as ‘just-in-time-migration’, this reframing aimed at making the right workers with the right qualifications available at the right time, without infringing the socially accepted leading principles in migration policy.

A second shift concerns the debate on working conditions for seasonal workers in agriculture. In general, the political debate on seasonal work in agriculture was very much focussed on the moment of perceived crisis in labour supply: in the 2 months between the entry ban and its end, 498 single argumentations regarding seasonal work were identified, with only 149 in the course of 2018 and 2019. In particular, the working conditions for seasonal labour in agriculture received increased attention. During the pandemic, several major outbreaks of the Corona virus among seasonal workers were documented, with several hundred people infected (LabourNet, 2020). The news of a seasonal worker on a farm in southern Germany who died on 11 April 2020 following a Corona infection caused particular alarm. The health risk posed by the Corona virus led to the precarious working conditions of seasonal workers being increasingly discussed in 2020. While in 2018 and 2019, almost exclusively the labour union IG BAU addressed the poor working conditions, since the beginning of the pandemic the parliamentary groups of the Greens, the Left, and the Social Democrats as well as the Ministry of Labour and Social Affairs have repeatedly pointed out structural abuses in occupational health and safety that were revealed by the pandemic and that needed to be changed. Thereby, problems that already existed in previous years, such as the lack of labour protection and its control, the circumvention of the minimum wage for seasonal workers or the disadvantages of social security exemption for workers, were increasingly acknowledged and addressed in the context of the pandemic. After the entry ban was lifted on 15 June, this attention quickly faded, as exemplified by only 19 mentions of seasonal work in 2 months from June to August 2020. Considering that the bigger part of COVID outbreaks and violations of occupational standards only became public after 15 June (LabourNet, 2020), the motivation for the political engagement with this topic clearly seems motivated by economic considerations, rather than the (persisting and worsening) dire working conditions. Still, later regulations like the obligation for employers to provide proof of their seasonal employees’ health insurances from 2022 or the stipulation of annual labour control rates for agricultural businesses (5% of all businesses) in the ‘Occupational Health and Safety Control Act’ adopted in December 2021 were informed by these shifts.

## Conclusion

This research aimed to empirically trace and inform political economic perspectives on seasonal labour migration on the macro-level to draw a more nuanced picture of its function within economic and migration regimes. Taking the moment of the pandemic as focal point and approaching the seasonal labour migration regime with an argumentation analysis of the political discourse allows to track negotiations and logics within the regime that before remained implicit.

As I have shown, the legal regime of seasonal labour is tailored to the needs of the agricultural labour market in terms of just-in-time migration, to make the right amount of migrant labour with the right characteristics available at the right time. The analysis also highlighted mechanisms of differential inclusion, showing the differentiation between seasonal workers, permanent EU migrants, asylum seekers and German nationals within the migration regime. While certain migrants shall be included temporarily in low-wage jobs without access to social rights (certain asylum seekers, temporary migrants), while other groups (Germans, permanent migrants) are deemed unfit for such jobs or meant to stay excluded altogether (asylum seekers from ‘safe countries’). Most importantly then, seasonal migrant labour functions as outsourced labour on the German labour market, temporarily taking up unwanted but essential positions on the labour market, while their reproduction costs, in particular social security entitlements, remain abroad. On the discursive level, seasonal work is produced as the ‘wanted’ form of migration by linking sufficient seasonal work migration as an economic interest of farmers to the interest of the ‘homeland’ and the necessary food supply for the German population. While the seasonal labour regime remained rather constant in these regards, discursive shifts could be observed at the moment of the pandemic: on one hand, working conditions received increased attention during the months of the entry ban. On the other hand, seasonal workers were suddenly referred to as ‘skilled workers’ to mark them as economically beneficial migration in accordance with German migration policy.

Altogether, seasonal workers seem to be the perfect migrants: they are the migrants with the right characteristics, coming for the right time without causing costs to social security or for their ‘integration’ while invisibly contributing to the emblematic German quality standards. Coming back to the puzzling advocacy for an increase of seasonal labour migration by right-wing actors I pointed to at the outset, their lack of opposition appears less surprising in the light of these insights: as just-in-time migration and outsourced labour seasonal workers only enter temporarily are not perceived as threat to the welfare state or nationalist identities. On the contrary, in the figure of the seasonal migrants the economic need of agricultural labour force merges with nationalist interests in protecting the ‘homeland’ and excluding migrants from the welfare state.

However, beyond the political perspective on the regime that has informed this article, the pandemic also transformed seasonal workers resistance and organising in struggling for their rights. Several strikes among seasonal workers arose since Spring 2020 and seasonal workers’ documentation of their working conditions initiated major debates in countries of origin on the exploitation of their nationals on German fields that prompted governmental representatives to act. This was the case for Romanian workers in 2020 (Cosma et al., 2020), as well as for Georgian workers in 2021 (stern, 2022), challenging the imaginary of third countries as a new reservoir for workers more willing to accept the given working conditions. The future will show, how migrants’ struggles – in combination with states’ increased consciousness to depend on seasonal workers– will shape seasonal labour migration regimes in the long run.

## Notes

1. This observation is shared by the workers union IG BAU and counselling services, who report very little attention for the situation of seasonal workers before the pandemic, notwithstanding annual awareness raising campaigns by a small group of workers unions, academia and activists (Initiative Faire Landarbeit, 2020; informal interview with workers union and counselling service).

2. The government, the Ministry of Agriculture and some parliamentary groups claimed that ‘this [extension] means that seasonal workers who have entered the country can stay longer’ (German Ministry of Agriculture, 2020d). Quite to the contrary, however, EU workers can legally stay and work in Germany independently from this regulation – only after this period employers need to pay social security contributions.

3. This information stems from an informal interview held with a representative of the advocacy organisation DBV.

4. As the experiences of counselling services (Initiative Faire Landarbeit, 2020; informal interview with counsellor) and ethnographic research suggests (Carlotta von Bock und Polach, 2011), the majority of seasonal workers in German agriculture come to Germany for several seasons and do not stay in Germany for much longer than the working period.

5. The workers union IG BAU and partially the opposition parties the Lefts and Greens explicitly addressed seasonal labour migrants as ‘essential workers’ (Die Linke, 2020; IG BAU, 2020: 3) to advocate for an additional payment for seasonal workers and increased labour protection. However, these measures were not adopted and the framing of the farmers as ‘essential’ to legitimise the measures taken (or demanded) dominated throughout the political discourse (on the notion of ‘essential workers’ see the contribution by Casas-Cortes & Cobarrubias (2023) in this issue).
